# Prevalence of allergic and nonallergic rhinitis in a rural area of northern China based on sensitization to specific aeroallergens

**DOI:** 10.1186/s13223-018-0299-9

**Published:** 2018-11-21

**Authors:** Yanran Huang, Yuan Zhang, Luo Zhang

**Affiliations:** 1 Department of Otolaryngology Head and Neck Surgery, Beijing TongRen Hospital, Capital Medical University, Beijing, China; 2 Beijing Key Laboratory of Nasal Diseases, Beijing Institute of Otolaryngology, No. 17, HouGouHuTong, DongCheng District, Beijing, 100005 People’s Republic of China; 3 Department of Allergy, Beijing TongRen Hospital, Capital Medical University, Beijing, China

**Keywords:** Allergic rhinitis, Atopic status, Epidemiologic study, Nonallergic rhinitis

## Abstract

**Background:**

Most epidemiologic studies reporting prevalence of allergic rhinitis (AR) and nonallergic rhinitis (NAR) have assessed solely self-reported prevalence, without confirmation by objective measures. Furthermore, reports of prevalence of NAR in Chinese subjects are scarce. Thus, we aimed to explore the prevalence and risk factors of AR and NAR in a Chinese, based on both clinical manifestation and allergic status.

**Methods:**

We conducted a population-based cross-sectional survey, involving 1084 local residents from a rural area of Beijing, China. Participants were enrolled using a stratified two-stage cluster sampling method. All adult participants or the guardians of children completed standardized questionnaires to provide relevant demographic and clinical information. Skin prick tests were also performed to determine sensitization to specific aeroallergens. AR/NAR was classified according to Allergic Rhinitis and its Impact on Asthma criteria.

**Results:**

Prevalence of self-reported AR was 46.80%. Based on SPT results, the confirmed standardized prevalence of AR and NAR were 16.78% and 24.60%, respectively. Severity scores for nasal itching, sneezing, rhinorrhea and congestion were significantly higher in subjects with AR, than subjects with NAR (*P *< 0.05 for all). The three most common aeroallergens in self-reported AR group were *Blattella germanica* (16.6%), *Dermatophagoides farinae* (14.6%), and *Dermatophagoides pteronyssinus* (13.9%). Family history of AR and atopic dermatitis were significantly associated with AR (adjusted OR: 4.97 and 2.69, respectively), whereas family history of AR and asthma were significantly associated with NAR (adjusted OR: 3.53 and 2.45, respectively). Similarly, comorbid asthma, CRS, and atopic dermatitis were significant risk factors for both AR and NAR.

**Conclusions:**

Combination of standardized questionnaires and specific allergen tests may provide more accurate estimates of prevalence of AR and NAR and associated risk factors.

## Background

Traditionally, rhinitis is divided into two types according to its etiology; allergic rhinitis (AR) and nonallergic rhinitis (NAR). Nowadays, most epidemiologic studies of AR are based on mostly self-reported prevalence of symptoms and often lack objective measures. Moreover, epidemiologic evidence of NAR is scant. Thus, we aimed to perform a population-based cross-sectional study in a rural community of northern China, to assess the prevalence of clinical AR and NAR, based on both subjective and objective measures.

As an inflammatory disease of nasal mucosa, rhinitis is often defined by its clinical manifestation, such as rhinorrhea, sneezing, nasal congestion and itching. Although sometimes considered as a trivial disease, because it is not associated with mortality, it can lead to a great financial burden and tremendously impair a patient’s quality of life; negatively impacting many aspects of life such as sleeping, working performance, emotion, socializing, etc.

According to the latest Allergic Rhinitis and its Impact on Asthma (ARIA) guidelines [1], classic symptoms of AR include sneezing, rhinorrhea, nasal congestion, and nasal pruritus, alongside with ocular symptoms such as redness and itching of eyes and lachrymation. Detectable immunoglobulin E (IgE) against relevant aeroallergens is also measurable, as the symptoms of AR are a result a hypersensitivity reaction caused by specific inhalant allergens, mediated by specific immunoglobulin E (IgE).

Globally, AR affects 10–40% adults and 2–25% children [2]. Evidence indicates that prevalence of AR is high and increasing throughout the world; with 23–30% in Europe [3], 12–30% in the United States [4], and 11.1–17.6% in China [5, 6]. According to several relevant epidemiologic studies, the prevalence of self-reported AR varies from 10 to 50% [6–13]; likely because of the coexistence of AR and NAR and many studies have evaluated the prevalence of AR solely based on questionnaire surveys, telephone interviews and self-reported symptoms. Objective tests such as skin prick test (SPT) and serum specific IgE were often not included in the data, leading to a higher prevalence. Indeed, one recent study by Zhang and the colleagues [11] to investigate the prevalence of allergic rhinitis (AR) and its associated risk factors in over 4000 3–5 years old preschool children in Beijing has reported that while the self-reported prevalence of AR was 48%, the prevalence of clinical AR, diagnosed based on SPT results, was 14.9%.

NAR, on the other hand, is a group of heterogeneous diseases, characterized by clinical nasal symptoms with negative specific allergen tests [14]. It is based on exclusive diagnosis, being nonatopic with nasal symptoms in response to inhalant and irritant triggers in the absence of a specific cause [14]. Although the mechanisms underlying NAR remain unclear, it is generally thought that innervation of the nasal mucosa may be involved [14–16]. In recent years, there has been renewed interest in NAR with the prevalence reported to be 7% in the United States [17]. However, there’s little consensus on the diagnostic criteria and lack of specific tests, and NAR is thus often misdiagnosed as AR.

Few epidemiologic studies have been reported on NAR, and most of the studies have concentrated solely on the self-reported prevalence. The aim of the present study was therefore to assess the prevalence of clinical AR and NAR, using both subjective and objective tests, and the associated risk factors in Chinese subjects from a rural community in Beijing, China.

## Methods

### Study design

This was a population-based cross-sectional observational study, conducted from November 2011 to December 2011. With a computer-generated list, the participants were recruited from four randomly selected communities in a randomly selected town in Huairou district, a northern rural district of Beijing, China, and directed to assemble at the regional medical care center. All volunteers, who formed half of the population in the selected region, completed a specifically designed questionnaire, as detailed below, under supervision of a group of experienced interviewers, and were also subjected to SPT for assessment of sensitization to specific inhalant allergens. All the children’s questionnaires and informed consent forms were completed by their guardians.

**Fig. 1:**
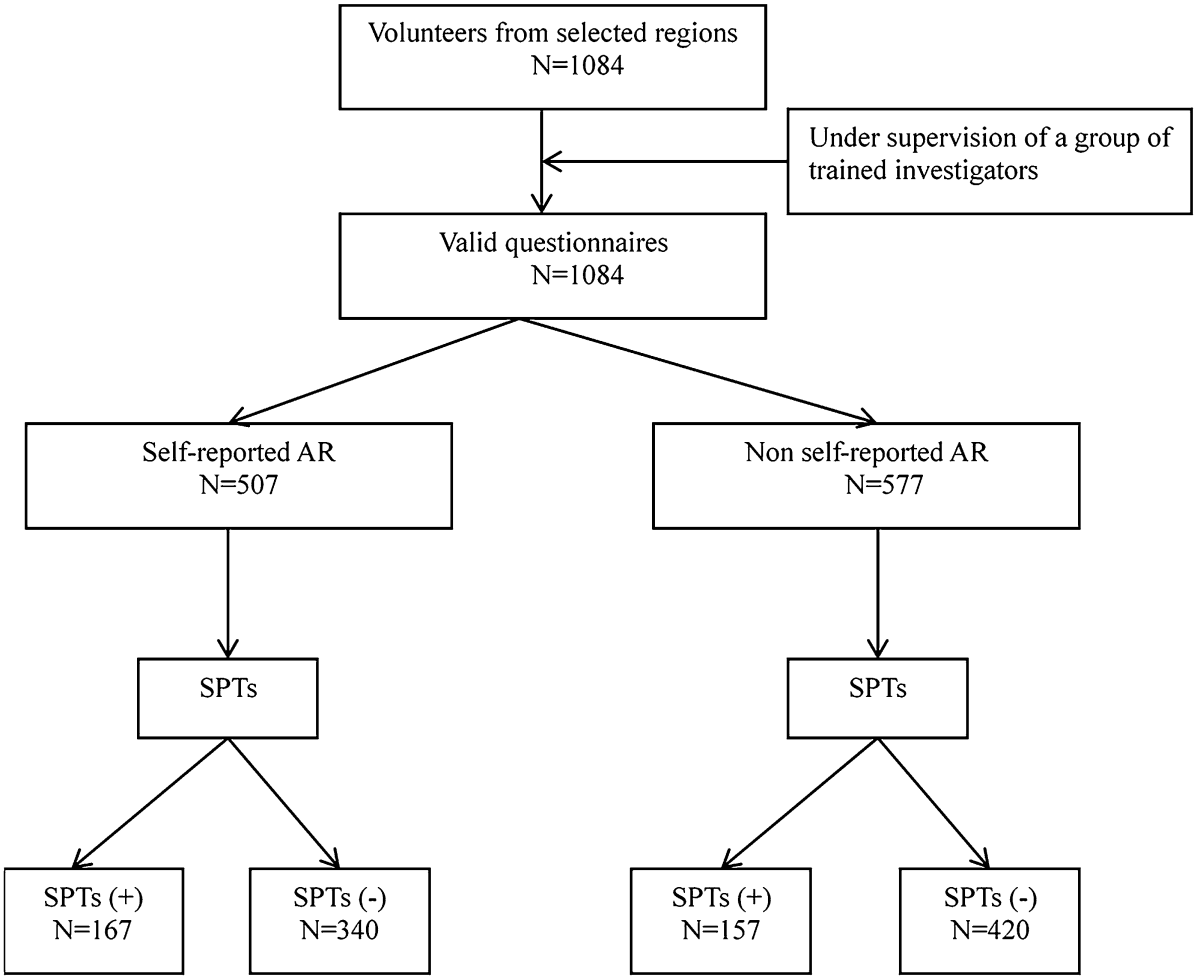
Flow diagram of the study. SPT, skin prick tests

The study protocol, as shown as Fig. 1, was approved by the ethics review board of the Beijing TongRen hospital and Beijing Institute of Otolaryngology, P. R. China; and written informed consent was obtained from all participants before enrolment into the study.

### Sample estimation

In order to assure sufficient statistical power for the calculation of clinical prevalence and better evaluation of epidemiological features, the sample size N was estimated before the study according to the formula 1 [18, 19]:

1 $${\text{N}}\, = \,{\text{Z}}^{ 2} {\text{P}}\left( { 1{-}{\text{P}}} \right)/{\text{d}}^{ 2}$$

where Z equaled to 1.96 as the level of confidence of 95%; d was chosen to be 0.03 as precision; P was estimated to be 0.2 based on the published epidemiological data of AR in China. As a result, the target sample size equaled to 683. Considering the sampling bias, the sample was set to be not less than 1000.

### Questionnaire design

The questionnaire comprised 24 questions to provide information on a subject’s general demographic characteristics (age, sex, occupation, financial situation, education, etc.), as well as information on self-reported rhinitis, duration and severity of rhinitis, ocular symptoms, nasal symptoms, duration and severity, self-reported allergens, other allergen-related diseases (chronic sinusitis, asthma and atopic dermatitis), smoking history and family history of allergic disease. The severity of symptoms and quality of life (QoL) were assessed by validated Chinese version visual analogue scale (VAS) and the rhinoconjunctivitis quality of life questionnaire (RQLQ) [20].

AR was diagnosed in accordance with the criteria of the 2010 ARIA guideline [21], and subjects were defined as self-reported AR patients if they had more than two of the four following typical symptoms of rhinitis; including sneezing, rhinorrhea, nasal congestion and itching in the past 12 months, excluding the effect of upper respiratory infections. Self-reported AR subjects were further classified into two groups based on the results of SPT; with the subjects showing positive SPT defined as clinical AR patients, and the subjects with negative SPT defined as clinical NAR patients. All AR patients were further classified into intermittent and persistent AR, based on the self-reported duration of nasal symptoms. Intermittent AR was defined as AR symptoms less than 4 days per week or less than 4 weeks per year, while persistent AR was defined as AR symptoms for more than 4 days per week and more than 4 weeks within the past 12 months. Based on the self-reported quality of life, this was further classified as mild or moderate-severe AR, according to the presence or absence of items including sleep disturbance, impairment of daily activities, sports, work or school performance, etc. Thus, AR was categorized as mild AR in the absence of any of these items, and as moderate-severe AR in the presence of one or more of these items.

### SPTs

SPT were performed by a group of specialized technicians, according to a standardized protocol. All the participants attended the study without taking any antihistamines or topical/systemic steroids for at least 72 h prior to SPTs; using a panel of 20 standardized aeroallergen extracts (Allergo pharma GmbH & Co. KG, Reinbek, Germany). The 20 aeroallergens tested included a mixture of animal hair (hamster, dog, rabbit, cat and guinea pig), tree, grasses, grass/cereals, mugwort, dandelion, giant ragweed, *Chenopodium album*, humulus, locus, *Blattella germanica*, pine, plantain, *Candida albicans*, *Penicillium notatum*, *Alternaria tenuis, Aspergillus fumigatus, Dermatophagoides farina* (*Der f*) *and Dermatophagoides pteronyssinus* (*Der p*). Histamine and saline were used as a positive and a negative control, respectively. The skin reaction towards each allergen was measured as the diameter of the wheal produced after 15 min. The result was based on skin index (SI = mean size of allergen weal/size of histamine wheal) [22]. In the study, SI ≥ 0.5 was confirmed to be a positive SPT result.

### Statistical analysis

Data were analyzed using SPSS V.22 software package (IBM Corp., Armonk, NY, USA). The regional study populations were standardized by age and gender of the reference population, based on the sixth China population census in 2010. Descriptive statistics were used for demographic and general information of the study population. Chi-square analysis was performed to analyze the clinical evaluation and the common sensitized allergens evidenced by SPT. Univariate analysis was used, followed by the multivariate logistic regression, to evaluate associated factors of clinical AR and NAR. Both the OR and the 95% CI were measured. A value of *P *< 0.05 was considered to be significant.

## Results

### Demographic and survey information of the study population

The demographic characteristics of the study population are shown in Table 1. A total of 1084 local residents, aged 5–68 years old, in Huairou, were enrolled into the study using a stratified two-stage cluster sampling method. Overall, 405 (37.36%) were male and 679 (62.64%) female; with 57.56% of the participants being 45–60 years old. The individual household income per year was classified into 7 grades based on “The 2008 Statistics Yearbook in China” published by National Bureau of Statistics of China, and showed that most of the volunteers were from minimum income households (283; 26.11%) and moderate-income households (112; 10.33%).

**Table 1:** Demographic and clinical characteristics of the study population

	Total N = 1084 n (%)	Clinical AR N = 167 n (%)^a^	Clinical NAR N = 340 n (%)^b^	Non self-reported rhinitis N = 577 n (%)^c^
Gender				
Male	405 (37.36)	68 (40.72)	139 (40.88)	198 (34.32)
Female	679 (62.64)	99 (59.28)	201 (59.12)	379 (65.68)
Age (years)				
≤ 14	56 (5.17)	9 (5.39)	19 (5.59)	28 (4.85)
15–29	99 (9.13)	14 (8.38)	20 (5.88)	65 (11.27)
30–44	294 (27.12)	59 (35.33)	67 (19.71)	168 (29.12)
45–60	624 (57.56)	82 (49.10)	232 (68.24)	310 (53.73)
> 60	9 (0.83)	2 (1.20)	1 (0.29)	6 (1.04)
Yearly income (Chinese yuan)				
Minimum (< 4604)	283 (26.11)	46 (27.54)	18 (5.29)	37 (6.41)
Low (4604–9568)	67 (6.18)	12 (7.19)	14 (4.12)	39 (6.76)
Below-moderate (9568–12,978)	65 (6.00)	12 (7.19)	30 (8.82)	71 (12.31)
Moderate (12,978–17,684)	112 (10.33)	11 (6.59)	24 (7.06)	56 (9.71)
Above-moderate (17,684–24,106)	89 (8.21)	9 (5.39)	24 (7.06)	32 (5.55)
High (24,106–40,019)	68 (6.27)	12 (7.19)	19 (5.59)	21 (3.64)
Maximum (> 40,019)	52 (4.80)	12 (7.19)	18 (5.29)	37 (6.41)
Medical history				
Asthma	40 (3.69)	13 (7.78)	20 (5.88)	7 (1.21)
Atopic dermatitis	99 (9.13)	28 (16.77)	50 (14.71)	21 (3.64)
CRS	71 (6.55)	21 (12.57)	35 (10.29)	15 (2.60)
Family history				
AR	85 (7.84)	30 (17.96)	39 (11.47)	16 (2.77)
Asthma	44 (4.06)	8 (4.79)	24 (7.06)	12 (2.08)
Atopic dermatitis	53 (4.89)	16 (9.58)	23 (6.76)	14 (2.43)
CRS	45 (4.15)	9 (5.40)	22 (6.47)	14 (2.43)
Smoking habit				
Smoker	191 (17.62)	32 (19.16)	74 (21.76)	88 (15.25)
Non-smoker	833 (76.85)	132 (79.04)	257 (75.59)	489 (84.75)

CRS, chronic rhinosinusitis

^a^Self-reported AR subjects showing positive skin prick test, ^b^ self-reported AR subjects with negative skin prick test, ^c^ non self-reported rhinitis subjects without symptoms of rhinitis

### The prevalence of clinical AR and NAR

Table 2 shows the prevalence of the clinical diagnosed AR and NAR. Overall, the prevalence of self-reported AR and clinical AR as defined in the present study were 46.77% and 15.41%, respectively, while the prevalence of clinical NAR as defined in the present study was 31.37%. After standardization for age and gender, the prevalence of clinical AR and clinical NAR were found to be 16.78% and 24.60%, respectively.

**Table 2:** The prevalence of clinical AR and NAR

	N	Clinical AR^a^			Clinical NAR^b^		
		N	Prevalence (%)	Standardized prevalence	N	Prevalence (%)	Standardized prevalence
Male	405	68	16.79	15.63	139	34.32	27.92
Female	679	99	14.58	16.76	201	29.60	23.75
Total	1084	167	15.41	16.78	340	31.37	24.60

^a^Self-reported AR subjects showing positive skin prick test, ^b^ self-reported AR subjects with negative skin prick test

The majority of clinical AR patients belonged to minimum income household (46; 27.54%) (Table 1). Asthma or atopic dermatitis alone were reported by 40 (3.69%) and 99 (9.13%) of the subjects, respectively, while the prevalence of comorbid asthma or atopic dermatitis was much higher in subjects classified as clinical AR patients (asthma, 7.78%; atopic dermatitis, 16.77%) and clinical NAR patients (asthma, 5.88%; atopic dermatitis, 14.71%). Similarly, family history of atopic disease (AR, asthma, atopic dermatitis), was reported by 7.84%, 4.06% and 4.89%, of all participants; which compared with 17.96%, 4.79%, 9.58% patients, respectively, in clinical AR group, and 11.47%, 7.06%, 6.76% patients, respectively, in clinical NAR group.

### Clinical evaluation

More subjects were classified as “intermittent” than “persistent” in both self-reported AR group (57.67% vs 42.33%) and clinical AR group (51.95% vs 48.05%). Similarly, patients with moderate-severe AR were more common in both self-reported and clinical diagnosed AR groups (72.48% vs 27.52%, 79.25% vs 20.75%, respectively). With no statistical significance observed between the two groups, both the self-reported and clinical AR groups shared the similar pattern of the duration and severity of the disease with no statistical differences (*P* = 0.091 and 0.215, respectively).

Both AR and NAR patients tended to suffer from severe nasal symptoms nasal itching, congestion and rhinorrhea, and moderate sneezing. However, as shown in Table 3, the nasal symptoms of clinical AR group were greater than the clinical NAR group (*P *< 0.05).

**Table 3:** Nasal symptoms and RQLQ scores of AR and NAR patient groups

	Clinical AR^a^	Clinical NAR^b^	*P value*
Nasal symptoms, VAS (mean ± SD)			
Itching	4.46 ± 3.50	3.09 ± 2.99	< 0.0001
Sneezing	1.53 ± 2.66	0.90 ± 0.94	< 0.0001
Rhinorrhea	4.29 ± 3.41	3.01 ± 2.95	0.004
Congestion	3.99 ± 3.29	3.42 ± 3.06	0.021
RQLQ (mean ± SD)			
Sleep	4.57 ± 4.61	3.17 ± 3.76	0.019
Non nasal/eyes	8.8 ± 9.08	6.11 ± 7.29	< 0.0001
Practical problems	4.92 ± 4.81	3.04 ± 3.77	0.459
Nasal	6.89 ± 5.94	4.57 ± 4.76	< 0.0001
Eyes	4.77 ± 5.76	2.91 ± 3.83	0.041
Emotion	5.38 ± 5.74	3.73 ± 4.79	< 0.0001

VAS, visual analogue scale; RQLQ, the rhinoconjunctivitis quality of life questionnaire

^a^Self-reported AR subjects showing positive SPT defined as clinical AR patients, ^b^ self-reported AR subjects with negative SPT

All the participants finished the RQLQ. The QoL of patients in both clinical AR and clinical NAR groups was impaired in all aspects; however, the clinical AR group showed significantly greater negative impact of all aspects, except disturbance from practical problems, which impaired the QoL of these patients.

Table 4 shows the sensitization aeroallergens (based on SPT results). The three most common aeroallergens in self-reported AR and non self-reported rhinitis (asymptomatic) groups were *Blattella germanica* (16.6%, 14.9%), *Der f* (14.6%, 10.9%), *Der p* (13.9%, 9.0%), respectively. Significantly greater numbers of subjects in self-reported AR group were sensitized to *Der f* and *Der p*, as well as to mugwort*, Penicillium notatum*, animal dander, dandelion, ragweed, and *Curvularia lunata*, than subjects in asymptomatic group. In the self-reported AR group, 9.5, 7.3, and 16.0% of the subjects were sensitized to one, two and ≥ three allergens. Similarly, in the asymptomatic group 9.8, 6.6, and 10.7% of the subjects were sensitized to one, two and ≥ three allergens.

**Table 4:** Common sensitizing allergens evidenced by SPT

	Self-reported AR N = 507^a^	Non self reported rhinitis (asymptomatic group) N = 577^b^	*P*-value
*Der f*	74 (14.6%)	63 (10.9%)	< 0.05
*Der p*	70 (13.9%)	52 (9.0%)	< 0.01
Mugwort	35 (6.9%)	22 (3.8%)	< 0.01
*Blattella germanica*	84 (16.6%)	86 (14.9%)	0.453
*Candida albicans*	2 (0.4%)	1 (0.2%)	0.073
*Penicillium notatum*	14 (2.8%)	12 (2.1%)	< 0.01
Animal dander	10 (2.0%)	0 (0.0%)	< 0.01
Tree	34 (6.7%)	29 (5.0%)	0.100
Grasses/Cereals	21 (4.2%)	21 (3.6%)	0.351
*Alternaria tenuis*	4 (0.8%)	3 (0.5%)	0.604
Dandelion	34 (6.7%)	18 (3.1%)	< 0.01
*Chenopodium album*	30 (5.9%)	22 (3.8%)	0.063
Ragweed	39 (7.8%)	28 (4.8%)	< 0.05
Locust	25 (5.0%)	18 (3.1%)	0.091
*Curvularia lunata*	9 (1.2%)	3 (0.5%)	< 0.05
Plantain	15 (3.0%)	12 (2.1%)	0.218
*Aspergillus fumigatus*	3 (0.6%)	2 (0.3%)	0.444
*Humulus*	8 (1.6%)	5 (0.9%)	0.207
Pine	8 (1.6%)	6 (1.0%)	0.297
Positive SPT			
To one allergen	48 (9.5%)	57 (9.8%)	
To two allergens	37 (7.3%)	38 (6.6%)	
To three or more allergens	81 (16.0%)	62 (10.7%)	

*Der f, Dermatophagoides farinae*; *Der p, Dermatophagoides pteronyssinus*; SPT, skin prick test

^a^Self-reported AR based on symptoms according to ARIA guideline, ^b^ non self-reported rhinitis subjects without symptoms of rhinitis

### The risk factors associated with clinical AR and NAR

In order to filter out the risk factors associated with clinical AR and NAR, all the associated factors were analyzed using Chi square, and the factors found to be significant (*P* < 0.05), were further analyzed using logistic regression test and multivariate analysis. In this regard family history of AR and atopic dermatitis were significantly found to be associated with clinical AR (adjusted OR 4.97 and 2.69, respectively) and family history of AR and asthma significantly associated with NAR (adjusted OR 3.53 and 2.45, respectively) (Table 5). Similarly, presence of comorbid asthma, chronic rhinosinusitis (CRS), and atopic dermatitis; but not cardiovascular disease or smoking; were significant risk factors for both AR and NAR (Table 5).

**Table 5:** Factors associated with clinical AR and NAR

	Clinical AR^a^		Clinical NAR^b^	
	*P*-value	OR (95% CI)	*P*-value	OR (95% CI)
Gender				
Male			NS	
Female			NS	
Family history				
AR	< 0.001	4.97 (2.45, 10.09)	< 0.001	3.53 (1.79, 6.96)
Asthma			< 0.05	2.45 (1.02, 5.87)
CRS			NS	
Atopic dermatitis	< 0.05	2.69 (1.14, 6.37)	NS	
Comorbidity disease				
Asthma	< 0.01	4.79 (1.57, 14.67)	< 0.01	4.36 (1.61, 11.85)
CRS	< 0.001	5.16 (2.37, 11.26)	< 0.01	3.21 (1.52, 6.78)
Atopic dermatitis	< 0.001	3.55 (1.78, 7.08)	< 0.01	2.75 (1.44, 5.25)
Cardiovascular disease			NS	
Smoking			NS	

NS, not significant; CRS, chronic rhinosinusitis

^a^Self-reported AR subjects showing positive skin prick test, ^b^ self-reported AR subjects with negative skin prick test

## Discussion

China, a country of diverse regions with a huge population of 1.3 billion, provides a wide-ranging patient base for investigating the epidemiology of rhinitis. Despite, a progressively increasing prevalence of AR over the last few decades [5] and availability of this large patient base, there is a marked paucity in epidemiological data; particularly the prevalence of AR and NAR in rural Chinese populations. Moreover, most of the available studies have reported their findings for prevalence of AR purely on the basis of the presence of self-reported nasal symptoms; with very few studies taking into account the importance of using objective tests for providing more accurate estimates, because of the cost and the low compliance of the subjects. In this respect Wang and colleagues [23] have reported the prevalence of AR in the northern China to be 9.2% when both the nasal symptoms and the result of sIgE were considered. Another study by Zhang and colleagues [11] combining a typical medical history of AR symptoms and diagnostic skin prick tests (SPT) reported that the prevalence of self-reported AR in Chinese children was 3.26 greater than clinically diagnosed AR. Indeed, one early nationwide study using telephone interviews for assessing prevalence of AR across China demonstrated that the prevalence of self-reported AR varied from 8.7% in Beijing to 24.1% in Urumqi (southwestern of China) [6]. It is possible that the wide variation in prevalence of self-reported AR varied might lie in the difficulty of understanding the questionnaires and the coexistence of NAR, which was not specifically addressed. Furthermore, it is possible that the variability may additionally result from differences in both training and comprehension of the disease by investigators in different centers, as adopting face-to-face interviews has shown the prevalence of AR to vary from 6.24% [13] to 32.5% [7]. In this respect, no comparative epidemiological data have been reported for prevalence of NAR in China. To our knowledge the present study is the first to provide such epidemiological data on both clinically diagnosed AR and NAR in a rural area of northern China, using a combination of self-reported AR and objective assessment of sensitization to specific aeroallergens by SPT.

Our study has demonstrated that the prevalence of clinical AR was 15.41%, which is similar to an AR prevalence of 16.67% found in a population-based survey of AR in Quebec in 2008 [24]. Moreover, the prevalence of NAR was 31.37% based on typical nasal symptoms as well as negative SPTs results. Similarly, age- and gender-standardized prevalence of AR was 16.78% and of NAR was 24.60%. Studies from the US and Europe have reported the prevalence of NAR to range from 7 to 19% in adults [17, 25, 26]. A study by Rondón and colleagues [27], involving a cohort of 428 randomly selected Spanish patients attending an allergy service, has further reported that local allergic rhinitis (LAR), which has a similar clinical profile to AR in nonatopic patients, is a distinct and prevalent entity, as indicated by diagnosis of 63.1% patients with AR, 11.2% with NAR and 25.7% with LAR. Thus, it is possible that in the absence of specific nasal provocation tests some subjects with local allergic rhinitis (LAR) may be misdiagnosed as NAR patients.

While two studies have previously reported the prevalence of self-reported AR in the rural areas of northern China to be between 6.2 and 10.8% [7, 23], another more recent multicenter study has indicated that the prevalence of self-reported AR was significantly increased over a 6-year period from 11.1% in 2005 to 17.6% in 2011 [9]. Indeed, we have previously have previously compared the prevalence of self-reported and skin prick test-confirmable AR among adults in urban and rural areas of China, and demonstrated that the prevalence of confirmable AR was similar between rural and urban areas in China, although there was a higher prevalence of self-reported AR in rural areas [7]. In the current study prevalence of epidemiologic self-reported AR was found to be 46.77% compared with 15.41% (16.78% after standardization for age and gender) clinical AR. These findings are in accordance with the findings of Zhang and colleagues [11], who also demonstrated prevalence of epidemiologic and adjusted clinical AR in preschool children in Beijing to be 48% and 14.9%, respectively. It is possible that the relatively high prevalence of self-reported AR in the present study compared to other studies in China [7, 9, 28] may partly be related to the socio-economic background of many of the participants, who were villagers with very low income and poor educational background, and found it difficult to fully understand the questionnaires, even under supervision of the investigators. Nevertheless, the present study indicated that although both clinical AR and NAR patients had severe nasal symptoms, which exerted negative effects on their QoL, the severity of the nasal symptoms in AR patients was significantly greater than in NAR patients.

Sensitization patterns for AR have been shown to vary from place to place, and are influenced by climate change [29, 30], gender [31], age [32, 33], etc. Race may also play a role because studies in Chinese [8, 34] and Korean [33] AR patients have shown dust mites to be the most prevalent sensitizing allergens, whereas studies in Europeans have pollen as the prevalent sensitizing allergens [3]. In the present study, *Blattella germanica* and the two house dust mites (*Der p* and *Der f*) were found to be the most prevalent allergens responsible for clinical AR, which is consistent with the findings of Lou and colleagues [8]. Indeed, these findings are also in agreement with the findings of a previous study, in which we demonstrated that *Blattella germanica, Der p, Der f,* as well as *Altenaria tenuis* were the most common sensitizing allergens in a cohort of over 10,000 AR out-patients [35]; and therefore suggest that a combination of standardized questionnaires and specific allergen tests may provide accurate and reliable estimates of prevalence of AR (and NAR) in population-based surveys.

Whilst *Blattella germanica* has been suggested to be an important component in the screening panels for aeroallergens in self-reported AR in most regions of China [8], there is limited evidence on the direct relationship between the sensitization of *Blattella germanica* and clinical manifestation of AR. Furthermore, although it is possible that the high sensitization rates to *Blattella germanica* noted in the present study may possibly be a result of cross reactivity with IgE for *Der p* or *Der f*; a study by Sun and colleagues [36] investigating the prevalence of sensitivity to cockroach allergens and IgE cross-reactivity between cockroach and house dust mite allergens in Chinese patients with allergic rhinitis and asthma seems to suggest that this is unlikely to be the case. These authors demonstrated that while there was a relatively high prevalence of cockroach sensitization in mainland China, the levels of cockroach SPT reactions were relatively low. Furthermore, although a large majority of cockroach sensitized patients were also SPT positive to *Der p*, an IgE cross-inhibition study showed that only a small number of patients appeared to have *Blattella germanica* and/or *Der p* as primary sensitizing source, which could false cause false positive SPT reactions against cockroach. However, the finding from the present study that sensitization rates for *Blattella germanica* in self-reported AR and NAR subjects were comparable (16.6% vs 14.9%) suggests that clinical manifestation of disease, especially the symptoms, should be compared with the specific allergen tests, rather than base a diagnosis of AR purely on tests in the clinical practice.

In this study, we found that family history of AR, and comorbid asthma, CRS, or atopic dermatitis, were all associated with increased risk of both AR and NAR. Although, it is somewhat surprising that a comorbid atopic disease, such asthma or atopic dermatitis, may confer a risk for development of a noninfectious, nonallergic condition such as NAR, this finding is nevertheless in accordance with the findings of Håkansson and colleagues [37] in adults and Chawes and colleagues [38] in children. Although the precise mechanisms underlying this association are not clear, it is possible that the “united airway” hypothesis [37, 39] or presence of a link between upper and lower airways beyond allergy associated inflammation [38] may, at least in part, explain this association. It is possible also that NAR may induced by to exposure to nonspecific triggers leading to nasal hyperreactivity [17], possibly in addition to or following development of AR and asthma. However, this would need to be confirmed in large longitudinal studies of patients and families with histories of allergic airways disease and NAR.

However, the findings of the present study are somewhat limited and thus need to be confirmed in further studies in consideration of these limitations, as well as in centers in other regions across China. In particular, in the present study NAR was differentiated AR, based only on the presence of typical rhinitis symptoms in the absence of negative SPT results, without data of IgE. In this respect, it is likely that the observed prevalence of NAR was higher than the actual prevalence because under the defined criteria for NAR in the present study patients with LAR would have been undistinguishable. Indeed, a systemic review of studies on AR or NAR patients subjected to diagnostic local nasal provocation has recently demonstrated that 26.5% of patients previously considered non-allergic demonstrate local allergen reactivity [40]. Moreover, the latest position paper of the European Academy of Allergy and Clinical Immunology on NAR [17] has indicated that NAR patients should be distinguished from AR patients with an allergic reaction confined to the nasal mucosa (i.e. LAR patients), probably by detection of nasal specific-IgE and/or positive nasal allergen provocation test (NAPT), although the latter is time consuming. Also, we have adopted the SI index for reporting the positive skin prick test size in this study; whereas a positive SPT is commonly reported as at least 3 mm greater than the negative control. Although it is possible that the low rate of positive skin prick tests noted in the present study may be due to the use of SI index, it is likely, however, that this finding is accurate for this study cohort; particularly because it included nearly half of the population of the region investigated and most of the subjects were healthy individuals.

## Conclusions

In summary, the confirmed standardized prevalence of AR and NAR were 16.78% and 24.60%, respectively. Combination of standardized questionnaires and specific allergen tests may provide more accurate estimates of prevalence of AR and NAR and associated risk factors.

## Abbreviations

NAR: nonallergic rhinitis
ARIA: Allergic Rhinitis and its Impact on Asthma
IgE: immunoglobulin E
SPT: skin prick test
QoL: quality of life
RQLQ: the rhinoconjunctivitis quality of life questionnaire
*Der f*: *Dermatophagoides farina*
*Der p*: *Dermatophagoides pteronyssinus*
SI: skin index
CRS: chronic rhinosinusitis
